# “I’m not alone”: perinatal women’s experiences in an online self-directed program for perinatal anxiety

**DOI:** 10.1186/s12884-025-07270-3

**Published:** 2025-02-21

**Authors:** Light Uchechukwu, Madison P. Hardman, Isabelle Hadley, Megan E. Gornik, Sarah K. Petty, Teaghan A. M. Pryor, Gillian M. Alcolado, Patricia Furer, Kristin A. Reynolds

**Affiliations:** 1 Department of Psychology, University of Manitoba, 190 Dysart Rd, Winnipeg, MB Canada; 2 Department of Clinical Health Psychology, University of Manitoba, Winnipeg, MB Canada; 3 Department of Psychiatry, University of Manitoba, Winnipeg, MB Canada

**Keywords:** Anxiety, Perinatal, Prenatal, Postpartum, Qualitative, Online program, Self-directed, CBT

## Abstract

**Background:**

Anxiety is highly prevalent during pregnancy and postpartum, and access to treatment can be difficult due to a range of barriers (e.g., time, distance, and service availability). Online treatments have the potential to circumvent these barriers and may, therefore, be beneficial for the perinatal population. The present study leveraged qualitative methods to understand participants’ perspectives on their use of a six-module online self-directed Cognitive Behavioral Therapy (CBT) program for perinatal anxiety as part of a randomized controlled trial.

**Methods:**

A mixed qualitative method design was used for this study. A total of 95 perinatal women were randomized to an intervention or waitlist control condition for an online self-directed program (*Overcoming Perinatal Anxiety*; OPA). Both waitlist and intervention participants provided open-ended feedback on each module via online surveys. A subset of individuals (*n* = 20) assigned to the intervention condition completed a virtual qualitative interview about their experiences using the program. Data obtained from open-ended survey questions and qualitative interviews were analyzed using Conventional Content Analysis (open-ended survey) and Reflexive Thematic Analysis (interviews).

**Results:**

Open-ended survey data were categorized into three themes, with associated sub-themes: *User experience* (subthemes: accessibility and modality), *Perceptions of content* (sub-themes: validating, informative, hopeful, anxiety-inducing, emotionally “heavy”, and helpful), and *Barriers to program engagement* (subthemes: lack of time and energy, technical difficulties, challenging and external factors)*.* Qualitative interview data were categorized into the following main themes, with associated subthemes: *Tensions in* e*ngaging with the self-directed program* (subthemes: connecting and multi-tasking, “finding the time,” module length and pacing, pen to paper, and “thanks for the reminder but don’t rush me”), *“I’m not alone,”* (subthemes: relating to the content, sharing anxiety with “inner circle,” and voicing a desire to connect with other “moms feeling the same way”), and “*I’m managing my anxiety”* (subthemes: “understanding my anxiety,” using “strategies to help with my anxiety,” “taking time for myself,” and moving forward).

**Conclusion:**

Findings highlight that online self-directed treatment can be an acceptable and feasible option for perinatal anxiety. Findings show promise for the scalability of OPA to improve access to psychological treatment for perinatal people experiencing anxiety.

**Trial registration:**

Clinical Trial Identifier: NCT04844138 (clinicaltrials.gov).

Trial registration submitted: [April 5, 2021] accepted: [April 14, 2021].

**Supplementary Information:**

The online version contains supplementary material available at 10.1186/s12884-025-07270-3.

## Introduction

The perinatal period spans conception to one year following childbirth [1]. This period is accompanied by an impactful lifestyle, physical, hormonal, and emotional changes [2, 3]. A qualitative study by Walker and colleagues [3] highlighted how substantial life changes during the perinatal period, such as altered eating habits, less physical activity, and reduced sleep, can negatively impact mental health. Along with these lifestyle changes, there are significant physical changes during this period, such as neural plasticity (i.e., maternal plasticity [4]). Such changes may alter brain functioning concerning motivation, threat detection, and emotional regulation [5], which may increase perinatal women’s vulnerability to developing mental health problems, such as anxiety [4]. Risk factors for perinatal anxiety include difficult pregnancy or delivery experiences, such as an emergency cesarian section; unplanned pregnancy; being a single parent; and a history of psychiatric disorders [6]. Taken together, these changes can lead to a new onset or worsening of anxiety symptoms, with research showing that one in five women in the perinatal period experience at least one anxiety disorder [7]. Therefore, it is crucial that effective treatments for perinatal anxiety be available.

Physical, mental, and lifestyle changes during the perinatal period are often accompanied by worries. Though some level of worry or anxiety is natural, it becomes concerning when worries are excessive, hard to control, and interfere with daily functioning [5, 8, 9]. Worries in the perinatal period may focus on specific parenting-related themes, such as worries about the health and safety of one’s growing fetus or infant, childbirth, and parenting [10, 11]. Anxiety is highly comorbid with depression in the perinatal population [12, 13], compounding this population’s need for timely access to treatment. Although the prevalence of anxiety disorders during the perinatal period is greater than mood disorders [7, 14], the literature concerning perinatal mental health has predominately focused on this population’s experiences with depression. This high prevalence in combination with the large number of annual births in Canada (351,679 in 2022 [15]) indicates that access to specialized anxiety treatment is critical.

Further, untreated anxiety symptoms have been shown to be associated with adverse maternal, fetal, and infant impacts. For instance, prenatal anxiety increases the vulnerability of developing postpartum depression [16] and is associated with a shorter gestational period [10]. Perinatal anxiety is associated with a reduced likelihood of breastfeeding engagement and maintenance [16]. Considering the impacts on the child, perinatal anxiety has been found to be a predictor of poor social-emotional development in children ages two and older [17] and has been associated with negative emotionality and internalizing and externalizing behavior [18]. Given these potential negative impacts, it is essential that appropriate and accessible treatment options are available to women during the perinatal period.

### Help-seeking and barriers

Finding ways to provide treatment to perinatal women who are struggling with anxiety is imperative, especially considering the range of help-seeking barriers faced by this population. Under 40% of perinatal women report the intention to seek help for mental health problems, and around 50% report shame, fear, and embarrassment as reasons for not seeking help [19]. Additional barriers to seeking help include time, cost of treatment, distance to the service, childcare and service availability [20–24]. When perinatal women choose to seek help, they are often faced with long waitlists [21]. To circumvent this issue, perinatal women have reported pre-emptively waitlisting themselves for services in anticipation of their symptoms worsening as the perinatal period progresses [21]. As this is neither a sustainable nor desirable solution to improving access to perinatal mental health care, novel advancements are needed.

### Treatment options for perinatal anxiety

Though there are a multitude of barriers to seeking treatment for perinatal anxiety, effective treatments do exist, including pharmacotherapy and psychotherapy. Due to concerns from clinicians and perinatal women concerning the safety of medications, psychotherapy is typically favored [25]. In particular, cognitive behavioural therapy (CBT) has demonstrated efficacy in anxiety reduction during the perinatal period [26–28]. The CBT model of anxiety views thinking and behavioural patterns as contributing to emotional distress, with treatment targeting physiological symptoms and anxious thinking (e.g., worst-case scenarios, minimizing the positive) and avoidance to promote engagement with perinatal life and new learning of helpful and balanced thoughts [29]. Both Furer and colleagues’ [26] and Green and colleagues’ [30] group CBT interventions resulted in a reduction in anxiety symptoms among perinatal women with a large effect size. In both studies, participants expressed high treatment satisfaction for the perinatal-focused CBT interventions and authors reported high levels of treatment satisfaction. Providing access to CBT is crucial for perinatal women experiencing anxiety, however, this can be challenging due to service availability and cost. The COVID-19 pandemic saw an increase in anxiety and depression [31, 32] but factors such as lockdowns and high demand severely impacted access to mental health treatment [33]. Difficulties with access to mental health treatment can still be seen years after the pandemic. Many Western countries (e.g., Canada, United States, and Australia) are facing an increased demand for psychological services but lack the capacity to meet this demand [34–36], which affects service wait times and access to mental health services [37]. Receiving treatment in a timely matter is a vital concern for perinatal women given the rapid physical and mental changes that happen during this short period of time [21].

### eHealth services

Given the range of help-seeking barriers that may hinder access to CBT programming, eHealth and mHealth (mobile health) services (a novel form of health care that involves the transfer of health information, services and communication through technologies [1]) may help to provide effective mental health treatment to the perinatal population. eHealth and mHealth programs are considered effective forms of treatment for anxiety disorders among both non-perinatal [38] and perinatal populations [39–41]. Such programs may be an effective means of providing psychological services to a greater number of perinatal women due to its flexibility and accessibility.

To the best of our knowledge there has been one self-directed CBT program for perinatal anxiety that has been developed and evaluated [40, 41]. Loughnan and colleagues^’^ [40, 41] Australian MUMentum CBT program was geared towards pregnant and postpartum participants with depression and generalized anxiety disorder and included three sessions. The authors found that the online program had a moderate to large effect size for the reduction of anxiety symptoms, a non-significant effect on the reduction of depression symptoms in pregnancy and a large effect size for the reduction of anxiety and depression symptoms in postpartum participants. However, the MUMentum studies did not include a qualitative understanding of participant experiences with the program, which aligns with the current research landscape where there is a lack of understanding concerning perinatal participants’ experiences accessing an online program for perinatal anxiety.

### The present study

The *Overcoming Anxiety in Pregnancy and Postpartum* (OPA) e-Health/m-health program is based on an in-person workbook developed by Furer and Reynolds [26, 42]. The in-person and online versions of the program include six modules; however, the online version can be completed at participants' own pace at an approximate rate of one module per week. If modules were not completed in the week, a reminder email was sent out with a link to the program. Modules included psychoeducation, self-care, setting goals and facing fears, nurturing development with the baby, coping with negative thoughts and worries, and looking forward. The program included audio and visual information and worksheets for participants to complete in each module. The aim of this study was to understand pregnant and postpartum women’s experiences using an online self-directed CBT program for perinatal anxiety. Given the lack of qualitative research concerning the use of eHealth/mHealth self-directed CBT programs for perinatal anxiety, we sought to understand how women experienced the OPA online self-directed e-health/m-health program by using a mixed qualitative methods design: open-ended survey responses after each program module, and individual semi-structured in-depth qualitative interviews after program completion.

## Methods

### Procedure & participants

Data were collected between April 2021 to June 2022 within a randomized controlled trial (see Reynolds and colleagues [43] for preliminary positive results) evaluating the effectiveness, feasibility, and acceptability of the OPA online program (ClinicalTrials.gov, identifier: NCT04844138). See Reynolds and colleagues [43] for the results of the RCT and Kristjanson and colleagues [21] for a detailed outline of procedures. Participants were recruited for the trial through hospital clinics, community organizations, social media, and mainstream media. The study advertisement included a link to the OPA website as well as the research coordinator’s email such that interested individuals could reach out to express interest and ask questions. Ethics approval was obtained from the University of Manitoba Research Ethics Board, Fort Garry Campus and the Shared Health Research Impact Committee prior to recruitment.

Individuals were eligible for the OPA trial if they were pregnant or within 12 months postpartum at recruitment, were over 18 years of age, were experiencing symptoms of anxiety, resided in the province of Manitoba, had Internet access, and were fluent in English. Individuals experiencing current symptoms of a substance use disorder, psychotic disorder, bipolar disorder, and/or current suicidality were not eligible to participate due to the level of severity and need not matching the level of support provided by the intervention. All participants were provided with a mental health resource list, and resources were discussed with those who were ineligible for participation. For a detailed outline of our participant screening process, please see Kristjanson and colleagues [21].

Eligible participants were randomly assigned to the OPA intervention condition or a six-week waitlist condition. Participants were asked to complete a series of online surveys with closed and open-ended questions assessing their symptoms of perinatal anxiety (Perinatal Anxiety Screen Scale [PASS] [44]) and depression (Edinburgh Postnatal Depression Scale [EPDS] [45]), stress levels (Kessler 6-item Distress Scale [46] and Perceived Stress Scale [47]), prenatal/postnatal attachment (Maternal Antenatal Attachment Scale [48], and Maternal Postnatal Attachment Scale [49]), and maternal self-efficacy (Maternal Efficacy Questionnaire [50]) at the start of the program, end of the program, and one month after program completion. At the end of the program, participants were asked to rate their satisfaction with the OPA program (Treatment Satisfaction Measure [TSM] [42]). Participants also completed a brief survey at the end of each module, which included a module feedback measure, a shortened version of the PASS, and the EPDS. A $10 Amazon e-gift card was provided to participants after completing each set of surveys. We used quantitative methods to help contextualize our sample and included a demographic questionnaire, PASS, and EPDS quantitative measures in the study. Qualitative methods were used to understand participants’ experiences with the program. See Table 1 for a full description of all measures used in the study.

**Table 1:** Quantitative and Qualitative measures

Measures	Description of measures
Quantitative	
Demographic questionnaires	A subset of demographic questionnaire was used in the current study. Participants were asked to provide information on their age, ethnicity, education, marital status, current mental health treatment (medication or therapy), previous experiences with anxiety and/or depression, and perinatal status (i.e., pregnant or postpartum)
Perinatal Anxiety Screening Scale	The Perinatal Anxiety Screening Scale (PASS; [44]) was administered to participants prior to beginning the OPA program, following program completion, and at the one-month follow-up. The PASS is a 31-item scale assessing the severity of anxiety symptoms during the perinatal period. Each item is completed using a 4-point Likert scale, with response options ranging from 0 (*Not at all*) to 3 (*Almost always*)*.* Lower scores on the PASS indicate lower levels of anxiety severity (asymptomatic = 0–20, mild-moderate anxiety = 21–41, severe anxiety = 42–93) [51]. The PASS has been shown to have excellent reliability (*a* = .96), test–retest reliability, and convergent validity [43]
Edinburgh Postnatal Depression Scale	The Edinburgh Postnatal Depression Scale (EPDS; [45]) was administered to participants prior to beginning the OPA program, following program completion, and at the one-month follow-up. The EPDS is a 10-item self-report measure assessing symptoms of postnatal depression and is acceptable for use with pregnant individuals [52]. The scale is sensitive to change [45] and has excellent test–retest reliability [53]. Each item is completed using a 4-point Likert scale, with unique response options for each item (scores range from 0 to 3). Total scores of 12 or greater suggest the presence of depressive symptoms [45], and lower scores on the EPDS indicate lower levels of depression severity (asymptomatic = 0–6, mild = 7–13, moderate = 14 – 19, severe depressions = 19–30) [54]. For the study, the 10th item (suicidality) was removed, because individuals expressing suicidality were screened out of the study, due to ethics concerns regarding the ability to monitor suicide risk quickly
Qualitative	
Semi-Structured Interviews	Semi-structured qualitative interviews were conducted by trained research assistants via Zoom Professional. The questions in the interview were open-ended and asked about participants’ experiences in the program, program feasibility, and suggested improvements. Questions included: *What did you enjoy about the program? Why?*; *How did you feel about the engagement with the program?*; and *What did you dislike about the program?*. The qualitative interview guide has been published as a supplementary material (see Additional file 1). The interviews lasted 30–60 min, were audio-recorded, transcribed using Trint, and were de-identified and reviewed for accuracy by a research assistant. Final versions of each transcript were uploaded to Microsoft Word for qualitative analysis
Treatment Satisfaction Measure	The Treatment Satisfaction Measure [42] was developed to evaluate the OPA in-person group treatment program. Participants who completed the program were asked to respond to six closed and open-ended questions about their experiences with the OPA program. The TSM was used to assess the feasibility and acceptability of the online self-directed program. Two open-ended questions from the TSM were included in the present study: *What did you like about the “Overcoming Anxiety in Pregnancy and Postpartum” self-directed eHealth/mHealth program?*; and *Do you have any suggestions for improving this program?*
Weekly Feedback Questionnaire	To obtain feedback about each module’s content and associated practice sessions, participants were given a series of closed and open-ended items at the end of each OPA program module. One of the open-ended items was included for analysis within the present study (*Other feedback for this module’s content*) in order to understand participants’ experiences using the OPA program

The open-ended survey included responses from all participants (waitlist condition and intervention condition), while the semi-structured interviews included 20 participants from the intervention condition. Of note, waitlist condition participants completed the intervention following their waitlist period of six weeks. Semi-structured qualitative interviews were conducted between August 2021 to February 2022. The first 20 participants in the intervention condition were asked if they would like to participate in a qualitative interview about their experiences in the OPA program. If a person chose not to participate, the next person on the list was asked until 20 people accepted the invitation. Individuals who completed the semi-structured interview received a $15 Amazon e-gift card.

## Participants

In total, *N* = 95 eligible participants were randomized to the OPA intervention (*n* = 48) and waitlist control conditions (*n* = 47). Open-ended survey data was obtained from between 42 to 66 participants (depending on the survey item), and 20 participants assigned to the intervention condition completed semi-structured qualitative interviews. Overall, our sample was 32.40 years old (*n* = 95, *SD* = 4.21) for open-ended survey participants and 33.55 years old (*n* = 20, *SD* = 3.58) for interview participants, and all participants identified as a woman (*n* = 95, 100%). The majority of participants identified as being of European origins (open-ended survey: *n* = 69, 81.2%; interview: *n* = 15, 75.0%) and postpartum at the start of their enrollment in the trial (open-ended survey: *n* = 67, 70.5%; interview: *n* = 14, 70.0%). Demographic characteristics for the entire sample and the qualitative interview sub-sample are further illustrated in Table 2.

**Table 2:** Sociodemographic characteristics at baseline (interview *n* = 20; open-ended *N* = 95)

Characteristics	Interview M(SD) or n (%)	Open-ended M(SD) or n (%)
**Perinatal status**		
Pregnant	6 (30.0%)	28 (29.5%)
Postpartum	14 (70.0%)	67 (70.5%)
**Education**		
High school graduate	-	-
Some college/trade school	-	-
Graduated college/trade school	-	11 (11.6%)
Some university	-	7 (7.4)
University undergraduate degree	7 (35.0%)	42 (44.2%)
University graduate degree	8 (40.0%)	29 (30.5%)
**Ethnicity**		
Indigenous	-	6 (7.1%)
European	15 (75.0%)	69 (81.2%)
Central/ South/ Latin American	-	-
Asian	-	5 (5.9%)
Other	-	-
**Marital Status**		
Single	-	-
Common Law	-	17 (17.9%)
Married	18 (90.0%)	77 (81.1%)
**Previous anxiety or depression in lifetime**		
No	-	12 (12.6%)
Yes	19 (95.0%)	83 (87.4%)
**Current mental health medication or therapy**		
No	11 (55.0%)	51 (53.7%)
Yes	9 (45.0%)	44 (46.3%)
**Age (years)**	33.55 (3.58)	32.40 (4.21)
**Total sample size**	20	95

Data for the qualitative interview participants is displayed under the interview column. The total sample size is presented under the open-ended column

(-) = *n* < 5

### Mixed qualitative methods

For the study, we used mixed qualitative methods [55] employing conventional content analysis [56] and Reflexive Thematic Analysis (RTA) [57] to understand participants' experiences with the OPA program. Conventional content analysis was used for the open-ended survey responses and RTA was used for the semi-structured interviews. We used a separate approach where data collection and analysis were performed separately [55]. Following analysis, data were interpreted to identify ways in which the findings converge and diverge from each other. This approach helps to connect and build on each other to provide a holistic understanding of participants’ experiences with the online OPA program.

### Quantitative analysis

Descriptive analyses were conducted using the Statistical Package for Social Science ([SPSS] Version 29.0; IBM Corp). Quantitative findings concerning the effectiveness of the randomized controlled trial are currently in preparation and will be published elsewhere.

### Qualitative analysis

**Fig. 1:**
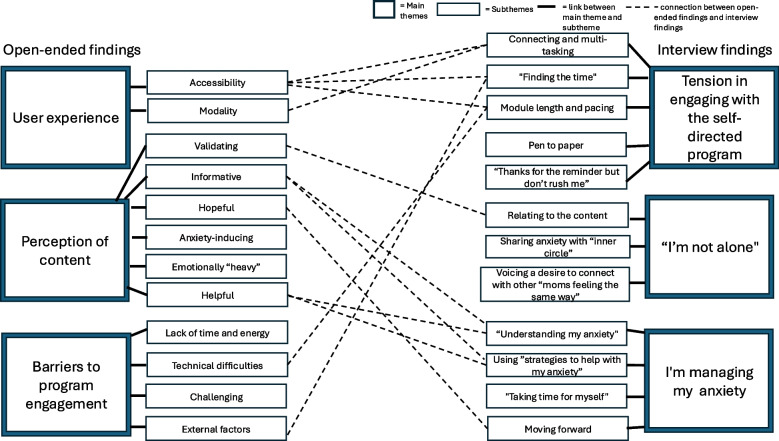
Integrated Thematic Map of Themes and Subthemes of Perinatal Women Perspective of a Self-Directed Online Program. Note. Open-ended findings a presented on the left, and interview findings are presented on the right. The Main themes are connected to the subthemes by a thick black line. Subthemes that have similarities between the two different findings are connected by a black dotted line

The open-ended survey and semi-structured interview data were analyzed separately by two researchers (IH and LU, respectively). While reviewing findings from the two separate analyses, we uncovered many overlaps between the two analyses. The overlap helps to demonstrate data triangulation, which helps to provide a more nuanced understanding of the data set [58]. A member of the research team (LU) combined findings from the short answer and interview data to form the result section of the current paper (see Fig. 1 for the integrated thematic map). Qualitative training was provided by members of the research team with expertise in qualitative methods and perinatal mental health (KAR, MPH). For the open-ended survey data, the study-coordinator (MPH) coded alongside the primary analyst (IH), and for the qualitative interview data, the PI (KAR) coded alongside the primary analyst (LU). Regular coding meetings for both analyses took place to discuss and refine thematic development.

A conventional content analysis was used to analyze the open-ended survey. Conventional content analysis is an inductive approach that requires researchers to immerse themselves in the data to understand participants’ experiences [56]. The conventional content analysis process includes deep immersion with the data, line-by-line coding by pulling out keywords and phrases, naming the codes, categorizing codes into themes and subthemes, and then defining the themes and subthemes [56]. All open-ended survey responses were coded using Microsoft Excel.

Reflexive Thematic Analysis (RTA) is a flexible and active process that requires immersed reflection and engagement with the data set [59]. Using RTA [57, 59, 60], we analyzed participants’ semi-structured interview transcripts. The RTA process includes familiarization with the data, systematic data coding, generating initial themes, developing and reviewing themes, refining, defining and naming themes, and writing the report [60]. The data was coded using an inductive approach, and all codes developed were close to the participants’ language. Semi-structured interview transcripts were coded using Microsoft Word.

### Reflexivity and rigor

Based on the study's exploratory aim, we began with an initial sample size of 20 participants. Following analysis of 20 interviews, we understood the themes and sub-themes to be fully describe, with a range of breadth and depth in rich experiences and quotations [61]. This aligns with Braun and Clark's RTA methodology which centers on gathering comprehensive data [62] which can show the complexity of participants’ experiences. When analyzing data, researchers followed guidelines for excellent qualitative research (e.g., rigour, sincerity, and self-reflexivity [58]). An analytic journal was maintained throughout all qualitative analyses with coding-related development, discussion, and changes made to the thematic framework. In RTA, two or more coders helps provide multiple perspectives and create a reflexive and collective process which adds “a richer more nuanced” examination of the data [59, pp594]. It is important to recognize how personal experiences can influence the data analysis process [58]. The lead analyst for qualitative interviews is a female psychology undergraduate student who immigrated to Canada at a young age. Though they were not pregnant or postpartum at the time of analysis, they immersed themself in perinatal mental health literature throughout this process while staying close to the data by immersing themselves in the familiarization stage to help better understand participants’ experiences. The thematic map included multiple perspectives including the undergraduate researcher, research coordinator, and research supervisor. The primary analyst for the open-ended survey data (IH) is a psychology undergraduate student with research experience in maternal mental health. Although they were not able to relate to the experience of pregnancy, they worked closely with the open-ended response data to clearly understand the participants’ experiences. The research coordinator (MPH) is a graduate student in Clinical Psychology with research experience in perinatal mental health and assisted with the development of the online program, screening of participants, and qualitative interviews. Given her pre-existing relationships with the participants, the research coordinator did not code any of the qualitative interview data but provided mentorship to coders if questions arose and was consulted on the development of the thematic framework. The research supervisor (KAR) is a Registered Clinical Psychologist with research and clinical expertise in perinatal populations and program development. The research supervisor conducted two qualitative interviews and provided mentorship, discussion, and review at all stages of the analysis.

## Results

Across the entire sample, participants’ average PASS scores were 38.03 (SD = 14.81), thus falling within the mild to moderate severity category. Their average EPDS scores were 12.03 (SD = 4.56), indicating that participants were in the mild severity category at baseline. Most participants had previously experienced symptoms of anxiety and/or depression (*n* = 83, 87.4%) Table 3.

**Table 3:** Qualitative quotes: open-ended survey questions

Main theme: User experience	
**Subtheme**	**Direct quote (participant ID)**
**Accessibility**	I like that you're able to go at your own pace, don't feel rushed to get it done so quickly! (Participant 93) [I] love[d] that they will read the text to you. (Participant 2)
**Modality**	The content was a great mix of information and exercises that have started my mental health journey at a time when it has been most needed. (Participant 96)
	Some redundancies in content. Could be more concise. (Participant 6)
	I really liked the practice assignments, the way the modules were organized, and that it was advised to space out the modules weekly. (Participant 95)
**Main theme: Perception of content**	
** Validating**	This module hit home. It made me feel less alone in the way I feel about situations and avoiding. (Participant 58) I felt good that others felt the same way and that I was not crazy. (Participant 29)
** Informative**	Informative, relevant, and useful. (Participant 60)
	I thought it was a good length and informative. (Participant 38)
** Hopeful**	Left me feeling very hopeful! (Participant 1)
	It’s giving me a lot of hope and courage for our next pregnancy in the future. (Participant 40)
** Anxiety-inducing**	The question in the beginning made me very stressed: my experience during COVID. I usually try not to think about it, but the question made me to think about it and it felt overwhelming. (Participant 72) Lots of goals to make, felt like homework and made me feel anxious. (Participant 68)
** Emotionally “heavy”**	I had a lot of emotions to work through. (Participant 1) I found it to be a bit workload heavy and emotion heavy. Lots of thinking and reflecting to be done back to back-to-back. (Participant 39)
** Helpful**	The content exploring perfectionism was very helpful. (Participant 107) I think completing the anxiety cycle was very helpful! It made me realize how everything is one never-ending cycle and also made me realize a few external triggers that I didn’t even realize affected me. (Participant 55)
**Main theme: Barriers to program engagement**	
** Lack of time and energy**	I guess I felt a bit of a time crunch to finish everything up because I think I'm supposed to finish within the week of it being sent out. (Participant 1) I am exhausted and feel like I don't have time for much. (Participant 79)
** Technical difficulties**	I don’t have a printer, and I would’ve liked hard copies of the documents. I would’ve loved to receive a booklet in the mail to work through. (Participant 63)
	I couldn’t download or save the assignments to my phone. I wish they were pdf documents. (Participant 19)
** Challenging**	Doing the goal setting was quite challenging. It took me a while to think about what I really wanted to achieve for myself/my life. (Participant 36)
	Goal setting was difficult for me. I felt like I needed to talk about it further or get some feedback or something to be able to see that goal as achievable. (Participant 94)
	I wasn't sure where to find the practice assignments. Were they in the modules? (Participant 5)
** External factors**	I had good intentions of completing the worksheets, and I fell very ill, as did my children. (Participant 48)
	Just life’s circumstances got in the way. Like a major move and parenting alone. (Participant 32)
	Hard with a baby and things opening up to do all the modules within a week. (Participant 42) It would not take me over three hours to complete normally…I just have to stop and tend to bab[y’s] needs.” (Participant 69)

### Qualitative findings: interviews

In the RTA analysis of the interview participants (*n* = 20)*,* three main themes were developed: *Tension in engaging with the self-directed program* (subthemes: connecting and multi-tasking, finding the time, module length and pacing, pen to paper, and thanks for the reminder but don’t rush me), *“*I’m not alone*”*(subthemes: relating to the content, sharing anxiety with “inner circle,” and voicing a desire to connect with other “moms feeling the same way”), and *“I’m managing my anxiety”* (subthemes: “understanding my anxiety,” using “strategies to help with my anxiety,” “taking time for myself,” and moving forward). Additional qualitative responses can be found in Table 4.

**Table 4:** Additional qualitative quotes: semi-structured individual interviews

Main theme: Engaging with the self-directed program		
**Subtheme**	**Direct quote (participant ID)**	**In-text identifier**
**Connecting and multi-tasking**	I did like how there was audio. So, I could kind of multitask at the same time. (Participant 74)	1
**Finding the time**	I like the online option because it was easier to access. It is easier with the baby to have something online that you can do at your own pace, and during your own time. Rather than having a scheduled meeting, because sometimes, that’s hard to navigate. (Participant 47)	2
	Maybe a bit of a barrier would have been finding the time. I would always try and schedule at least like an hour chunk of time, so that I could do the program by myself without any interruptions, because I found that was when it was helping me the most. (Participant 1)	3
	To do it every Monday, I feel like that's a lot. So much happens in your life, especially during pregnancy. (participant 65)	4
**Module length and pacing**	I think the pace worked really well. (Participant 46)	5
**Pen to paper**	In terms of being online versus in person, I think that, um, it was hard for me to do some of the exercises because I wanted to, you know, you want to write and scribble and do that, and they didn't have any the materials. I had to kind of, and I don't have a printer, so I had to kind of draw the tables and then fill them out, which was kind of onerous, you know? So, I would recommend maybe to allow people to either purchase a printed and mailed copy of those things, or to provide them for- for the course. (Participant 46)	6
**“**Thanks for the reminder but don’t rush me**”**	Getting that email, I felt rushed to get the next one done and, it didn’t feel…self-directed, like on my own time. (Participant 69)	7
**Main theme: “**I’m not alone**”**		
** Relating to the content**	I think what I enjoyed the most is that, even though it was about overcoming anxiety, the examples were specific to pregnancy and postpartum. So, it was easy to relate to and maybe think about things like, wow, I didn’t realize other people in postpartum have these kinds of difficulties too. (Participant 40)	8
** Sharing anxiety with “inner circle”**	[The program noted] these are some things you might experience, and I took a screenshot and sent it to my husband and… I was showing a lot of it to him about stuff that I was experiencing, and that was really helpful. (Participant 49)	9
	I was able to have a conversation with my inner circle, to be like, listen, I took this [program], these are my warning signs, if I do this, I need you to support me in this way. (Participant 65)	10
** Voicing a desire to connect with other “moms feeling the same way”**	Would have been wonderful if it was possible to have some kind of group with the people doing this, or some kind of anxiety support group for moms, specifically around pregnancy and birth. (Participant 47)	11
	If you are able to connect with other moms, then you can just hear their stories and how they're thinking, it validates what you're going through a little bit more. (Participant 86)	12
**Main theme: Managing my anxiety**		
** Understanding my anxiety**	When I'm having to work through some stuff, I'm realizing, okay, no, that's not a normal thought, this isn't normal behavior, this is anxiety. (Participant 1)	13
** Using “strategies to help with my anxiety”**	I like that it gives you exercises to do throughout the week and things to think about. (Participant 91)	14
	The most helpful part of the program was the goal setting area. It was nice to print off those PDFs and have the goals written down and then referring back to them. (Participant 26)	15
** “Taking time for myself”**	The really helpful one was the self-care week… I need reminders of that constantly, like, it's on the back burner. (Participant 46)	16
** Moving forward**	I took photo screenshots of some of the sheets so that I have them on my phone so that I can look back, and I printed out the forms so I can go back. (Participant 29)	17

Interview participants’ quotes that are not included in the body of the paper which can be found by the in-text identifier number

### Tensions in engaging with the self-directed program

The first main theme was categorized by participants' discussion of how they engaged with the self-directed program and the components, which both improved and hindered accessibility and engagement. Across this theme, the importance of program flexibility was noted by many participants, particularly during the busy periods of pregnancy and postpartum when there is never enough time.

*For me I would do it when my daughter was asleep, so I would do it at ten o’clock at night. For me, it was better and that was the time that was quiet... Which I wouldn’t get if it was in person.* [Participant 91]

Other program components that improved and hindered engagement with the program included information delivery. Participants could either listen to or read the information provided in addition to videos demonstrating certain exercises (e.g., deep breathing). Participants also discussed the module length, which included content and practice sessions, the program’s pace, and reminder emails’ impact on their engagement with the program. In this theme, the tension arises from participants’ enjoyment of the program’s format (e.g., flexibility, delivery of content and pace of the program) but still craving additional support to help with program engagement (e.g., more flexibility, physical workbook copy, and shorter modules). Therefore, the subthemes for this module included: connecting and multi-tasking, finding the time, module length and pacing, pen to paper, and thanks for the reminder but don’t rush me.

#### Connecting and multi-tasking

Participants voiced their appreciation for the video and audio options that the program provided. Participants explained how this was an important feature for their learning style. For example, Participant 91 stated, “For my personal learning, when I read, sometimes I just start straying and I'm not connecting to it, so I have to read it again, but for me when I see videos, I connect to it a little bit more.” Additionally, audio and visual options were a way for participants to complete the program while completing other tasks related to the baby or home (Table 4, quote 1).

#### Finding the time

Participants reported program-related advantages and disadvantages in discussing ‘time’ as it related to their participation in the program. The advantages were that they could work on the program on their own time, and many participants appreciated the flexibility the program provided (Table 4, quote 2). One participant described how she completed the program while taking care of her baby:

*So, I’d be just sitting there feeding baby, trying to stay awake and thinking, oh, no, and all the worries would roll in. Then I'd just pull out my phone, and I can hold the phone while I'm holding the baby, and I can go through the module and I can make little notes, and I can quiet down the thoughts, right? So that was, that was really helpful.* [Participant 25]

Even in this flexible, online self-directed program, time is still a prominent barrier that some participants faced, either in general or during certain periods (e.g., family, illness, holidays; Table 4, quotes 3 and 4).

#### Module length and pacing

It was recommended that participants complete one module per week. Although some participants liked the pace of the program (Table 4, quote 5), others reported wanting more time to do the modules or to sit with the material. One participant expressed a desire for the modules to be shorter in length, mentioning that she would rather do an additional module in exchange for a shorter module, “Sometimes the length of them did feel a bit long… I would have been ok with… an additional module” (Participant 71).

#### Pen to paper

Many participants reported difficulty with the PDFs provided and reported a preference to have a physical workbook such that they could write notes down on paper.

*Regardless of if I was on my phone using it or on my computer, I always made notes on my pad of paper. Because I'm old school, so to speak, I don't like making notes on the computer.* [Participant 91]

Some participants also expressed how having a workbook would have made it easier, especially those who didn’t have a printer and wanted to print out the worksheets (Table 4, quote 6).

#### Thanks for the reminder but don’t rush me

Participants were divided in their perceptions of helpfulness surrounding reminder emails, which were sent to participants when they did not complete a module during the previous week. Some participants noted that they appreciated the reminder emails in helping them stay on task.

*I didn’t find it was challenging to keep up, except for remembering to go back every week. So, I found the reminders helpful for that.* [Participant 74]

However, some participants reported that reminder emails added to their anxiety, (Table 4, quote 7).

#### I’m not alone

In the second main theme, participants described feeling less alone, through relating to the content and how the program helped facilitate communication with close others. In this main theme three subthemes were developed (relating to the content, sharing anxiety with “inner circle,” and voicing a desire to connect with other “moms feeling the same way”) that captured how the program facilitated in helping participants not feel alone.

#### Relating to the content

Participants reported that they related to the content and the examples given in the program materials (Table 4, quote 8). Program content helped them to understand their anxiety and feel less alone in their experience.

*Throughout different modules, there were always examples that were provided, and it helped me, be like, oh wow, if they have these examples in here and they're examples of what I'm genuinely feeling, obviously I'm not alone in these thoughts.* [Participant 1]

#### Sharing anxiety with “inner circle”

Participants described communicating about the program or their anxiety with loved ones (Table 4, quote 9). For example, Participant 62 discussed, “To actually learn what’s happening and to be able to put a name to it, with my husband so he understands where my head’s at… [it has] been pretty helpful.” Participants also discussed how the program helped to facilitate conversation with close others (Table 4, quote 10).

#### Voicing a desire to connect with other “moms feeling the same way.”

Participants also reported liking the idea of integrating group sessions into the program which would allow communication with other people in the perinatal period (Table 4, quote 11). Participants also expressed how hearing and sharing struggles with other moms were something they thought would been nice and would have helped them feel more validated (Table 4, quote 12).

*Based on the questions that I was being asked, I could safely assume that people were feeling the same way... But to hear it from other moms... I have the same worries. I don't get dressed either. Then you know that you're not the only person.* [Participant 69]

#### I’m Managing my anxiety

When so much of the focus of the perinatal period is on fetal and infant development, it can be challenging to find time to focus on one’s own health. Participants described the program as helpful in managing their anxiety, particularly concerning the connection between their thoughts and their anxiety. Participants also discussed how CBT strategies helped them manage their anxiety currently and make plans for the future. Accordingly, four subthemes were developed: “understanding my anxiety,” “using strategies to help with my anxiety,” “taking time for myself,” and moving forward.

#### Understanding my anxiety

Participants talked about how learning about their anxiety was one way that helped them manage their anxiety. By being able to recognize negative thoughts (Table 4, quote 13) and factors that lead to anxiety, participants felt better able to deal with their symptoms. One participant stated, “Learning especially about the cycle of it all, and how to recognize that it’s happening to me… helps me put a stop to it” (Participant 62).

#### Using “strategies to help with my anxiety”

One way participants managed their anxiety was by using the strategies provided in the program (Table 4, quote 14). Participants highlighted tools such as goal sheets, relaxed breathing, and progressive muscle relaxation exercises were (Table 4, quote 15). Participants described that they learned and used these skills throughout the program, and it was an important part of daily anxiety management. For example, one participant shared, “I was so much more aware of my anxiety and so much more aware of the strategies to help with my anxiety” (Participant 1).

#### Taking time for myself

Participants reported that the program helped them to take time for themselves.

*I am making time for myself, there are definite improvements. It did make me recognize I needed to make that time for myself and gave suggestions on how you can do that.* [Participant 31]

The program also helped participants focus on self-care which they found to help them with anxiety and stress (Table 4, quote 16).

#### Moving forward

Finally, participants reported that the program helped them manage their anxiety and the plans they had moving forward. Plans involved going back and reviewing the program and using strategies they learned from this program in the future (Table 4, quote 17). As mentioned by one participant “I think it gives me something I can still work with and work on um some of the stuff I learned, I can continue forward” (Participant 31).

### Data integration

Across the two qualitative data sets, there were many similarities helping to demonstrate data triangulation which provides a deeper understanding of participants’ experiences with the online program. Findings from both data sets indicated that participants enjoyed the program's flexibility, with participants voicing their enjoyment in completing the program on their own time and at their own pace. Findings also highlighted how external life circumstances (e.g., taking care of infants and older children at home; illness) affected program engagement and staying on track with homework exercises. Participants additionally reported benefiting from the content and format of the program while also recommending several improvements to enhance accessibility and engagement. These included a physical workbook to write in and fillable PDFs on the online system. Participants also enjoyed the multi-mode formats of receiving program content, including videos, text, and audio options to read program text. Participants expressed how the program was informative and how its content and strategies were helpful in their experiences being pregnant and/or postpartum with anxiety. Lastly, a prominent theme cutting across open-ended and interview analyses captured the validating and relatable nature of the program, which helped participants to recognize that they are not alone in their experiences.

The key difference between the two qualitative data sets was that different challenges in program engagement were presented. In the open-ended responses, participants raised some concerns with engagement with the program, such as difficulties with the practice assignment content, needing additional feedback, and trouble navigating the website to find practice assignments. Participants also expressed how some of the program’s content was anxiety-inducing and heavy, a topic that was not often mentioned in the interviews. Additionally, feelings of frustration and increased distress were mentioned by some participants during qualitative interviews, which were not presented in the open-ended survey responses, such as anxiety about reminder emails. The two data sets complemented each other, with the open-ended survey responses being more focused while the interview responses were more holistic. The interview responses offered deeper insight into some of the themes in the open-ended survey. Taken together, it is helpful to understand where findings converge and diverge, as well as the ways in which the two qualitative data sets present complimentary and also distinct and additive information, as this data will strengthen the revisions to OPA.

## Discussion

Overall, the study provides a novel contribution to the literature as it is the first study to our knowledge to explore perinatal women's experiences of an online CBT program for perinatal anxiety. One in five perinatal women experience an anxiety disorder [7], and as such, it is important to find ways to provide accessible treatment for this population. Online treatment programs offer the potential to reach a larger range of women and circumvent several key help-seeking barriers that impact this population (e.g., lack of childcare and time constraints). The aim of this study was to understand participants' experiences with the online perinatal anxiety self-directed program. Through open-ended surveys and semi-structured interviews, we were able to gain insight into participants' experiences with the eHealth/mHealth program. Participants faced some challenges with the program, such as difficulties with PDFs, feeling rushed, and needing more guidance with assignment sheets. Nonetheless, participants reported that they appreciated the flexibility of the program and the ways in which the modules were delivered. Despite the online and self-directed nature of the program, participants felt less alone in their experiences with anxiety, and the program facilitated connection with loved ones. They also appreciated the information and strategies provided in the program, as they helped them manage their anxiety. The result of this study highlights participants' acceptance of the OPA program as they found the program flexible, informative, and validating. It also helps to demonstrate ways in which the program can be improved.

In terms of engaging with the self-directed program, participants in both analyses described that they enjoyed the online format, particularly the flexibility that the program provided as it could be completed at any time. These findings align with previous studies highlighting perinatal women's desire to access flexible mental health treatments [23]. Research in the perinatal and general population has reported flexibility as being a benefit of online treatment, and some perinatal women have also reported a preference for online treatment over in-person [23]. Of note, even when allowing flexibility to work on the program in one’s own time, many participants still reported finding time as a barrier to program participation. Therefore, although the online format does not eliminate the time barrier altogether, it appears to be a helpful alternative for perinatal women. One consideration to enhance perinatal women’s ability to participate in treatment may be to increase access to childcare, as this may provide perinatal women with more time [63] which could be used to access mental health supports. Future research should investigate a randomized controlled trial with the OPA program that involves a childcare component to see if it helps to further alleviate the time constraint.

Participants expressed finding many aspects of the online program helpful in managing their anxiety, such as the information provided, strategies provided (e.g., deep breathing, muscle relaxation), and their plans to use them in the future. Learning about anxiety and understanding a person’s anxiety have been reported in perinatal and in the general population as important in dealing with their anxiety [64, 65]. Perinatal women have mentioned struggles in recognizing when they are having anxiety [23], noting that understanding their anxiety has helped them overcome it [64]. CBT also provides effective skills that a person can use for the rest of their lives [9]. In this study and other online CBT studies participants have expressed appreciation for the strategies provided and how they plan to use them in the future [66].

Another finding that had a conflicting impact on participants’ experiences was the reminder emails. Some participants appreciated the emails, while some found them to be anxiety-inducing. The negative impact of reminder emails is surprising based on previous research, which was found to contribute to increased motivation to complete a web-based intervention for adults with coronary heart disease and chronic pain [67]. Given the mixed findings concerning reminders, there appears to be room to improve this process, such as changing the wording of the emails to be softer and more inviting. Alternatively, these reminders could include a brief summary of the next module in the email to help motivate participants [21]. Future iterations of the OPA program could test different versions of reminder emails to see which emails support program completion.

Many participants emphasized a desire to write down program content on paper or receive a hard copy of the workbook. One limitation of the OPA program was that the PDF documents were not fillable, which led participants to print out copies of the PDFs; however, not all participants had the option to print out program materials. Other participants reported writing down notes about each exercise in a journal. Handwriting notes has been correlated with better quality notes and being more engaged with the information being given [68]. Therefore, it is important for future iterations of online CBT programs to consider the benefit of a physical workbook and to consider adding a physical copy alongside online material to future online programs.

Participants reported feeling less alone in their mental health experiences as a result of the OPA content. Importantly, feeling alone physically and psychologically is a factor that contributes to the development of perinatal anxiety [69] and has been correlated with worsening mental health [70]. Additionally, participants reported how the program helped them communicate with their partners and inner circle about their anxiety. In previous research, perinatal women have reported that communicating with partners, family, and friends had positive impacts on the perinatal experience and can be empowering [71, 72]. Nonetheless, participants also reported a need for more communication with other perinatal women, which aligns with previous qualitative research on this population [64]. There can often be unrealistic expectations concerning what the perinatal period should look like which can leave perinatal women feeling isolated [64, 72]. Therefore, creating spaces for perinatal women experiencing anxiety to connect may be beneficial, as such connections may help reduce anxiety symptoms [69]. Overall, findings suggest that eHealth/mHealth programs for this population may benefit from elements of peer support, which should be explored in future research.

Interestingly, participant discussion concerning COVID-19 was limited across interviews, and as such, no thematic content relating to COVID-19 was developed. A possible reason for this could have been the time the program was administered. The program was administered from April 2021 to June 2022. Around the end of 2021, Manitoba saw fewer cases of COVID-19 [73] which could have contributed to stress reduction. An example of how time affected stress levels regarding COVID-19 is Benatov and colleagues' [74] study, which measured stress levels at two different time points in a year and saw a reduction of stress at the second time point. Additionally, vaccines could have influenced the findings of the current study. By March 1st, 2021, 47,780 first doses of vaccine were administered to Manitoban citizens [75]. A study found that receiving the first dose of the vaccine reduced mental distress [76]. Time and vaccine might have been two possible explanations for why COVID-19 was not a main theme in the analysis.

### Limitations

Although this study is additive to the literature, we acknowledge that the perspective captured in the study consists of participants who largely identify with European heritage. Findings, therefore, may not reflect the experiences of perinatal people belonging to racial or ethnically diverse groups or members of the 2SLGBTQIA + community. Research on perinatal transgender and non-binary peoples’ experiences with mental health is scarce; however, the limited literature indicates that these groups often face feelings of isolation in the perinatal phase [77]. Future research should explore if an online self-directed program for perinatal anxiety would be similarly effective for transgender and non-binary people. Moreover, participants were experiencing mild to moderate levels of anxiety, which limits the transferability of findings to perinatal women experiencing more severe symptoms. Future research should explore whether individuals experiencing a broad spectrum of anxiety symptoms would find eHealth/mHealth programs for perinatal anxiety acceptable and or feasible with dealing with their anxiety. Additionally, all participants required access to a stable Internet connection, which could have acted as a barrier for certain groups to participate, such as low-income individuals. Future programs should consider offering a physical workbook with the online program. Incorporating the two components will not only appeal to individuals' different preferences but may be a way to provide treatment to those without Internet access. The study did not use a participant engagement framework, which may have helped to foresee some of the challenges participants expressed with the program (e.g., fillable PDF). Future researchers developing eHealth/mHealth programs may benefit from using a participant engagement framework and having their targeted demographic be actively engaged in the development of the program. Lastly, participants expressed a need to connect with other perinatal women. This lack of peer support is a common limitation among self-directed programming [66]. Future iterations of this program should look to partner with community organizations to deliver a hybrid version of the current program with a peer support component.

## Conclusion

The findings provide evidence for the acceptability of an online CBT program for perinatal anxiety. Participants expressed their acceptance of the program by highlighting how its flexibility supported their engagement with the program, which is important given the multiple competing demands present during the perinatal period. They also felt connected to the material provided and expressed hope for the future and plans to use the material provided in the program. Nonetheless, factors such as the program's pace and reminder emails affected engagement. Future iterations of the OPA program may employ shortened modules to help alleviate the feeling of being rushed, which may help to increase engagement.

Overall, our findings help to grow the literature on the acceptability of online self-directed programs. eHealth and mHealth programs are growing and have been used to treat mental health disorders in both the perinatal and non-perinatal populations. The study’s findings demonstrate the positive impacts that online self-directed programs can have on the provision of mental health services. Countries such as Australia, the United States, and Canada are experiencing excessive waitlists due to the high demand for mental health services, which is putting a strain on the healthcare system [34–36]. Online self-directed programs may help alleviate some of the stress on the healthcare system by providing mental health services to a vast number of people. This is especially crucial for the perinatal population in light of the negative impacts that untreated anxiety symptoms can have on both mother and child and the high prevalence of anxiety in this population. eHealth/mHealth anxiety program can be an acceptable way to access treatment for perinatal anxiety, but this study also highlights that treatment for perinatal anxiety is complex and should not be looked at through a one-size-fits-all-lens. eHealth/mHealth treatment should be offered along with an array of other treatment options (e.g., peer support, self-directed workbooks, group treatment) so that perinatal women can pick a treatment option that works best for them.

## Supplementary Information

Additional file 1. Individual Semi-Structured Interview questions Description of data: The file contains all questions asked to participants in the semi-structured interviews over Zoom.

## Data Availability

The datasets analyzed during the current study are not publicly available to protect participants' identity but can be available from the corresponding author on reasonable request.

## Abbreviations

CBT: Cognitive Behavioural Therapy
EPDS: Edinburgh Postnatal Depression Scale
K6: Kessler 6-item Distress Scale
OPA: Overcoming Anxiety in Pregnancy and Postpartum
PASS: Perinatal Anxiety Screening Scale
RTA: Reflexive Thematic Analysis
