# Supplementary material for: “I’m not alone”: perinatal women’s experiences in an online self-directed program for perinatal anxiety
Source: BMC Pregnancy Childbirth. 2025 Feb 21;25:190. doi: 10.1186/s12884-025-07270-3 (PMC11846167; doi:10.1186/s12884-025-07270-3)
Supplement: Supplementary file 1 — Additional file 1. Individual Semi-Structured Interview questions Description of data: The file contains all questions asked to participants in the semi-structured interviews over Zoom. [file 12884_2025_7270_MOESM1_ESM.pdf]

Additional file 1: **Individual Semi-Structured Interview Questions**

1. Tell me about your experience participating in the Overcoming Anxiety in Pregnancy and Postpartum online self-directed program.
    a. What did you enjoy about the program? Why?
    b. How did you feel about your engagement with the program? What motivated you to keep completing the modules? Were there any barriers you experienced?
    c. What did you dislike about the program? Is there anything that could be changed to address this?
2. Did your involvement lead to any important changes in your life? How so?
    a. How did the program impact your daily functioning?
3. How (if at all) did the program address your concerns around COVID-19?
4. What specific components of the program stood out to you? Why/how so? Are there any that you are still practicing?
5. Did you complete the practice sessions? How regularly? Did you find this helpful?
    a. What did you think about the workbook?
    b. What did you think about the format (i.e., electronic rather than paper)?
6. How did you experience the online program? (access to Zoom, interactions) Would you have preferred an in-person program?
7. How did you find navigating the online program.
8. How did you find the pace of the program? We recommending completing one module per week, is that what you ended up doing? Why or why not?
9. What can we do to improve the program going forward?

10. Now that you’ve completed the program, how are you doing with regards to your perinatal anxiety?
    a. Do you see yourself needing additional services to help with your anxiety? If so, do you have a plan for that?
11. Is there anything else you would like to add before we end the interview?

Probes were also used throughout:

- Can you tell me more about that?
- Can you provide me with an example of what you mean by that
